# Healthcare use before and after suicide attempt in refugees and Swedish-born individuals

**DOI:** 10.1007/s00127-020-01902-z

**Published:** 2020-06-16

**Authors:** Ridwanul Amin, Syed Rahman, Petter Tinghög, Magnus Helgesson, Bo Runeson, Emma Björkenstam, Ping Qin, Lars Mehlum, Emily A. Holmes, Ellenor Mittendorfer-Rutz

**Affiliations:** 1 Division of Insurance Medicine, Department of Clinical Neuroscience, Karolinska Institutet, 17177 Stockholm, Sweden; 2 Department of Global Public Health, Karolinska Institutet, 17177 Stockholm, Sweden; 3 Swedish Red Cross University College, 14157 Huddinge, Sweden; 4 Centre for Psychiatry Research, Department of Clinical Neuroscience, S.t Göran’s Hospital, Karolinska Institutet, Stockholm County Council, 11281 Stockholm, Sweden; 5 National Centre for Suicide Research and Prevention, University of Oslo, 0374 Oslo, Norway; 6 Department of Clinical Neuroscience (CNS), K8, Psychology, Karolinska Institutet, 17177 Stockholm, Sweden; 7 Department of Psychology, Uppsala University, 75237 Uppsala, Sweden

**Keywords:** Migration, Refugees, Attempted suicide, Cohort, Healthcare, Disability pension

## Abstract

**Purpose:**

There is a lack of research on whether healthcare use before and after a suicide attempt differs between refugees and the host population. We aimed to investigate if the patterns of specialised (inpatient and specialised outpatient) psychiatric and somatic healthcare use, 3 years before and after a suicide attempt, differ between refugees and the Swedish-born individuals in Sweden. Additionally, we aimed to explore if specialised healthcare use differed among refugee suicide attempters according to their sex, age, education or receipt of disability pension.

**Methods:**

All refugees and Swedish-born individuals, 20–64 years of age, treated for suicide attempt in specialised healthcare during 2004–2013 (*n* = 85,771 suicide attempters, of which 4.5% refugees) were followed 3 years before and after (Y − 3 to Y + 3) the index suicide attempt (t0) regarding their specialised healthcare use. Annual adjusted prevalence with 95% confidence intervals (CIs) of specialised healthcare use were assessed by generalized estimating equations (GEE). Additionally, in analyses among the refugees, GEE models were stratified by sex, age, educational level and disability pension.

**Results:**

Compared to Swedish-born, refugees had lower prevalence rates of psychiatric and somatic healthcare use during the observation period. During Y + 1, 25% (95% CI 23–28%) refugees and 30% (95% CI 29–30%) Swedish-born used inpatient psychiatric healthcare. Among refugees, a higher specialised healthcare use was observed in disability pension recipients than non-recipients.

**Conclusion:**

Refugees used less specialised healthcare, before and after a suicide attempt, relative to the Swedish-born. Strengthened cultural competence among healthcare professionals and better health literacy among the refugees may improve healthcare access in refugees.

**Electronic supplementary material:**

The online version of this article (10.1007/s00127-020-01902-z) contains supplementary material, which is available to authorized users.

## Background

Suicide attempts present major public health concerns, because the behaviour often initiates at an early age, is prone to repetition and may result in an adverse health and social development [1]. To overcome this public health burden, identification of vulnerable populations is important to plan preventative and rehabilitative strategies. In this regard, refugees need attention because of the higher risk of depression and anxiety disorders in this population [2, 3]. In turn, these disorders are among the main risk factors for suicidal behaviour [4, 5]. However, there is knowledge gap regarding whether risk of suicide attempt, compared with a country’s host population, is different for refugees [6, 7]. A recent cohort study in Sweden reported a lower risk of suicide attempt in refugees, compared with the Swedish-born population [7]. The knowledge gap in this field seems even more prominent in light of the increase in global migration in the recent decades.

With increasing migration, the need for adaptability of the healthcare system in the host country becomes more important. Here, the socio-cultural differences of migrant populations (including refugees) also shape their healthcare needs. On one hand, migrants’ access to healthcare is dependent, to some extent, on the cultural competency and communication skills of the healthcare professionals in the host country [8, 9]. On the other hand, culturally determined perceptions of healthcare and help-seeking behaviour may lead to differential healthcare use in refugees [9, 10]. Additionally, structural barriers such as location of the healthcare centre or the burden of healthcare expenses may exist [9]. In some studies, type and quality of healthcare and treatment have been reported to be different in refugees and other migrants, in comparison with the host population [11, 12]. Here, stigma towards both mental ill-health and suicidal behaviour may take shape according to the refugees’ origin culture and religion [10, 13].

There is a scarcity of research on potential differences concerning healthcare use before and after a suicide attempt between refugees and the host population. In studies where refugees and non-refugee migrants were not distinguished, a lower healthcare use was reported among migrants in comparison with the host populations [14, 15]. Compared with the respective host populations in Europe, a higher likelihood of recommending no care at all or non-psychiatric care to Non-European migrants after a suicide attempt was found [14]. Similar finding of lower psychiatric healthcare use following self-harm was observed in south Asian migrants in the UK, compared with the majority population [15].

Although a high prevalence of mental disorders in refugees has been reported [2, 3, 16], we hypothesised that specialised healthcare use might be lower in refugees, before a suicide attempt, compared with the Swedish-born population. This assumption is based on refugees’ [17] and migrants’ (including refugees) [18–21] healthcare use in general. While healthcare use before a suicide attempt provides crucial knowledge from a prevention perspective, healthcare use after a suicide attempt gives necessary information on adequate follow-up care. The National Board of Health and Welfare in Sweden recommends adequate and special follow-up care e.g. ASSIP (Attempted Suicide Short Intervention Program) for individuals who attempted suicide so that future suicidal behaviour can be avoided [22]. Under the national health insurance system, refugees who are granted residence permit in Sweden are entitled to the same access to healthcare as the majority Swedish-born population. Even then, compared with the Swedish-born, a lower healthcare use after a suicide attempt in refugees is hypothesised, owing to the aforementioned barriers [8–10].

Furthermore, previous studies have reported variability in migrants’ specialised healthcare use according to healthcare settings (inpatient vs outpatient healthcare) and type of healthcare (psychiatric vs somatic) [20]. Specialised psychiatric healthcare use is primarily interesting in relation to a suicide attempt. Likewise, in case of refugees, somatic healthcare use may carry complementary information regarding comorbidity. Somatic healthcare use, particularly the length of care, following a suicide attempt may also vary according to the method used, i.e. whether a violent method was used or not. Furthermore, idioms of distress in refugees may present as physical symptoms or somatisation, which in turn can influence their somatic healthcare use [23]. Refugees may also express somatic symptoms of underlying mental illness to the healthcare professionals due to culturally influenced stigma associated with mental ill-health [24].

The patterns of healthcare use before and after a suicide attempt are also likely to show considerable individual variation between refugees. These variations might be due to discrepancies in e.g. socio-demographic factors, such as sex, age and educational status. Psychiatric healthcare use was found to be varying between refugee women and men [25, 26] and according to age groups among migrants [18, 20]. Socioeconomic status measured as educational attainment is often considered as a determinant of healthcare use in migrants, although the evidence regarding this is inconclusive [20]. Similarly, variability in healthcare use among refugees may arise due to differential marginalisation in the labour market. Unemployment, sickness absence and disability pension altogether can be conceptualised as labour market marginalisation from a social insurance perspective [27]. Here, variability in healthcare use according to disability pension status is particularly interesting, because disability pension can be considered as the permanent marginalisation from the labour market, as compared to unemployment and sickness absence, which are temporary marginalisation. Capturing such differences in healthcare use according to socio-demographic and labour market marginalisation characteristics is, therefore, important for designing culturally sensitive preventive strategies.

## Aims

We aimed to investigate if the patterns of specialised psychiatric and somatic healthcare use, within an observation window of 3 years before and 3 years after a suicide attempt, differ between the Swedish-born population and refugees in Sweden. A secondary aim was to explore if patterns of specialised healthcare use differ among refugee suicide attempters according to their sex, age, education or receipt of disability pension.

## Materials and methods

### Study population

The study population comprised an open cohort of all individuals, 20–64 years of age with at least one hospitalisation or visit in specialised outpatient healthcare due to suicide attempt between January 1st 2004 and December 31st 2013 (*n* = 99,050 suicide attempters) in Sweden. The observation period regarding healthcare use was 3 years before and 3 years after the index suicide attempt. Individuals not residing in Sweden during the 3 years prior to the index suicide attempt were excluded (*n* = 2062). Moreover, those with incomplete information on their country of birth (*n* = 13) and reason for residence in Sweden (*n* = 3534) were excluded. Because the population of interest in this study comprises Swedish-born and refugees, non-refugee migrants (*n* = 7670) were not included. The final study population included 85,771 individuals, of which 81,916 were Swedish-born and 3855 (4.5%) were refugees.

### Refugees and the Swedish-born population

Following an asylum seeking period, individuals who are granted permanent residence in Sweden are identified as refugees in this study. The definition provided by the Geneva Convention on Refugees [28] is used by The Swedish Migration Agency to grant residence permits with the following ‘reasons for residence’: ‘refugee’, ‘in need of protection’ and, ‘humanitarian grounds’ [29]. A sensitivity analysis, including or excluding the latter two ‘reason for residence’ groups as refugees, showed similar results (data not shown). All individuals born in Sweden were identified as ‘Swedish-born’. Refugee status was measured in the year of index suicide attempt or in the year before that.

### Registers

Suicide attempts were identified from the National Patient Register (national coverage available from 1987 for inpatient and from 2001 for specialised outpatient healthcare). Individual data were linked to information from the following registers, using anonymised unique Swedish personal identity number:

1. Statistics Sweden: LISA database (longitudinal integration database for health insurance and labour market studies) [30] contains personal data on socio-demographic factors and labour market marginalisation characteristics such as sex, age, country of birth, educational level, family situation, type of residential area, number of annual net days with sickness absence benefits, disability pension and number of annual days with unemployment; STATIV database (longitudinal database for integration studies) includes data on reason for residence (e.g. refugee).
2. National Board of Health and Welfare: National Patient Register with data on date and diagnosis of inpatient and specialised outpatient healthcare; and the Cause of death register (data on date and cause of death) [31].

### Suicide attempts

In this study, suicide attempt was coded according to the International Classification of Diseases version 10 (ICD-10): Self-harm (ICD-10 code X60-X84) and events of undetermined intent (ICD-10 code Y10-Y34). In analogy with previous studies [32, 33], events of undetermined intent were included as suicide attempts in this study. This method has shown to reduce bias from underreporting and to ensure coherence in case ascertainment [32–34]. The inclusion of events of undetermined intent can be considered as a strength in refugee studies because the underreporting of suicide attempts might be considerably larger in refugees than in the host population, as refugees might be more inclined to refuse or hide their intents as a psychological defence mechanism or due to the fear of stigmatisation [7]. However, this inclusion may have introduced misclassification. Therefore, a sensitivity analysis was conducted excluding undetermined intent which showed comparability of the results. The first hospitalisation or visit in specialised outpatient healthcare due to a suicide attempt in the period 2004–2013 was referred to as the index suicide attempt. The inclusion of suicide attempters seeking healthcare in the specialised outpatient led to a different sex distribution (more men than women) in our cohort than what is generally found in this research field, i.e. more women attempts suicide than men [35]. For this reason, sensitivity analyses were carried out by excluding suicide attempters who sought healthcare in the specialised outpatient. These analyses showed similar results to our main analyses (data not shown).

### Outcome

Outcome measures comprised psychiatric and somatic healthcare use, measured separately from inpatient and specialised outpatient healthcare. First, we calculated crude prevalence rates defined as the proportion of individuals having had such healthcare (Yes/No) during each observation year (Y − 3 to Y − 1 and Y + 1 to Y + 3). Second, estimated prevalence of healthcare use was calculated after adjusting for several covariates. Finally, healthcare use was also conceptualised as the mean annual number of visits to inpatient or specialised outpatient healthcare and the mean annual duration (days) of hospitalisation among those who had such healthcare during each of the observation years. In case of overlapping spells of inpatient healthcare, days that overlapped were only counted once to calculate the total duration [36]. All healthcare use due to ICD-10 codes F00-F99 as the main diagnoses were regarded as psychiatric healthcare use. All other ICD-10 codes (except ‘F’, ‘O’, ‘P’ and ‘Q’ codes) as the main diagnoses were regarded as somatic healthcare use.

### Covariates

The following factors were considered: A. Socio-demographic factors (sex, age, educational level, family situation and type of residential area); B. Labour market marginalisation factors (unemployment, sickness absence, disability pension); C. Factors related to index suicide attempt (year of index suicide attempt, method of suicide attempt, history of any inpatient (during 1987–2003) or specialised outpatient (during 2001–2003) healthcare due to suicide attempt, mental disorder as main or secondary diagnosis in specialised healthcare at index suicide attempt). Socio-demographic covariates were measured as of 31 December of the year before the index suicide attempt. Labour market marginalisation factors were measured for the entire year before the index suicide attempt. Missing values for a covariate were categorised in separate categories. Table 1 shows the categorisation of sociodemographic and labour market marginalisation factors. Supplementary Table 1 shows the categorisation of the factors related to index suicide attempt.

**Table 1:** Socio-demographic and labour market marginalisation characteristics of 81,916 Swedish-born and 3855 refugees^a^, aged 20–64 years and residing in Sweden in the baseline year^b^ who sought inpatient or specialised outpatient healthcare due to a suicide attempt (index suicide attempt) in between 2004 and 2013 in Sweden

Characteristics	All, *n* (%)	Swedish-born, *n* (%)	Refugees, *n* (%)
	85,771 (100.0)	81,916 (95.5)	3855 (4.5)
Socio-demographic factors^c^			
Sex			
Women	40,716 (47.5)	38,933 (47.5)	1783 (46.3)
Men	45,055 (52.5)	42,983 (52.5)	2072 (53.7)
Age (years)			
20–24	19,678 (22.9)	18,838 (23.0)	840 (21.8)
25–34	19,472 (22.7)	18,427 (22.5)	1045 (27.1)
35–44	17,697 (20.6)	16,712 (20.4)	985 (25.6)
45–54	16,296 (19.0)	15,550 (19.0)	746 (19.4)
55–64	12,628 (14.7)	12,389 (15.1)	239 (6.2)
Educational level (years)			
Compulsory school (0–9)	22,730 (26.5)	21,356 (26.1)	1374 (35.6)
High school (10–12)	46,088 (53.7)	44,558 (54.4)	1530 (39.7)
College or university (> 12)	16,057 (18.7)	15,233 (18.6)	824 (21.4)
Missing	896 (1.0)	769 (0.9)	127 (3.3)
Family situation			
Married/cohabiting without children living at home	10,037 (11.7)	9,646 (11.8)	391 (10.1)
Married/cohabiting with children living at home	15,383 (17.9)	14,316 (17.5)	1067 (27.7)
Single^d^ without children living at home	54,312 (63.3)	52,281 (63.8)	2031 (52.7)
Single^d^ with children living at home	6039 (7.0)	5673 (6.9)	366 (9.5)
Type of residential area^e^			
Big cities	26,087 (30.4)	24,236 (29.6)	1851 (48.0)
Medium-sized cities	31,707 (37.0)	30,388 (37.1)	1319 (34.2)
Small cities/villages	27,977 (32.6)	27,292 (33.3)	685 (17.8)
Labour market marginalisation factors^c^			
Unemployed, 1–180 days^f^	14,193 (16.5)	13,296 (16.2)	897 (23.3)
Unemployed, > 180 days^f^	3311 (3.9)	2967 (3.6)	344 (8.9)
Sickness absence, 1–90 net days^g^	9315 (10.9)	9023 (11.0)	292 (7.6)
Sickness absence, > 90 net days^g^	8680 (10.1)	8390 (10.2)	290 (7.5)
Disability pension^h,i^	16,923 (19.7)	16,411 (20.0)	512 (13.3)
Index suicide attempts			
From inpatient healthcare	40,091 (46.7)	38,120 (46.5)	1971 (51.1)
From specialised outpatient healthcare	45,680 (53.3)	43,796 (53.5)	1884 (48.9)

Differences between the Swedish-born individuals and the refugees regarding all socio-demographic and labour market marginalisation factors were statistically significant based on Chi-square tests (*p* < 0.05)

^a^Individuals who settled in Sweden as ‘refugee’ or ‘in need of protection’ or, ‘humanitarian grounds’

^b^The year prior to the index suicide attempt

^c^Measured during the baseline year

^d^Single/divorced/separated/widowed

^e^Type of residential area: big cities—Stockholm, Gothenburg and, Malmö; medium-sized cities—cities with more than 90,000 inhabitants within 30 km distance from the centre of the city; small cities/villages

^f^‘No unemployment’ category is not presented

^g^‘No sickness absence’ category is not presented

^h^‘No disability pension’ category is not presented

^i^Individuals having a disability pension during the baseline year

### Statistical analyses

Differences in the distributions of baseline socio-demographic, labour market marginalisation characteristics and factors related to index suicide attempt among the suicide attempters were tested using the Chi-square test. The date of the index suicide attempt was defined as time point ‘*t*_0_’ and the 3 years of observation before and after t0 comprised Y − 3 to Y − 1 and Y + 1 to Y + 3, respectively. Annual crude prevalence rates of healthcare use (inpatient and specialised outpatient healthcare due to psychiatric or somatic diagnoses) were calculated in the entire cohort of refugees and the Swedish-born population and group differences were tested using the Chi-square test. Mean number of visits to specialised outpatient healthcare and mean duration and number of hospitalisations for a particular observation year were calculated only among those with such healthcare. The differences in these means between the refugees and the Swedish-born were evaluated by independent sample *t* tests.

Annual adjusted prevalence rates of specialised healthcare use with 95% confidence intervals (CI) were assessed using repeated measure logistic regression analysis with the generalized estimating equations (GEE) method and an autoregressive correlation structure [37]. All GEE models were adjusted for sex, age, educational level and year of index suicide attempt. Due to risk of over-adjustment in the GEE models, we could not adjust for differences in baseline labour market marginalisation between the Swedish-born and the refugees. Therefore, a sensitivity analyses was done in a cohort of suicide attempters who had no unemployment, sickness absence or disability pension in the baseline year. When specialised healthcare use was compared between the Swedish-born and refugees in this selected cohort, it generally did not differ between these groups during the observation window (Supplementary Fig. 1).

Additionally, GEE models were used to analyse the estimated annual adjusted prevalence of specialised healthcare use among the refugees, stratified by sex, age, educational level and receipt of disability pension at baseline (These covariates were mutually adjusted in the respective models). Healthcare use was considered as missing in the subsequent follow-up years for individuals who died or emigrated during Y + 1 and Y + 2 (*n* = 2917 individuals, of which 80 refugees). All analyses were conducted in SAS v. 9.4 except the GEE analyses were carried out in SPSS v. 25. For all analyses, a *p *value less than 0.05 was considered statistically significant.

### Ethics

Ethical approval was obtained from the Regional Ethical Review Board, Karolinska Institutet, Stockholm, Sweden (Dnr: 2007/762-31).

## Results

### Socio-demographic and labour market marginalisation characteristics

There were more men than women, both among refugees and among the Swedish-born population (Table 1). Compared with the Swedish-born, refugees had a lower proportion of 55–64 year old individuals, more individuals with 0–9 years of education, more individuals who were married/cohabiting with children living at home and more big-city dwellers (Table 1). A greater proportion of refugees than the Swedish-born were unemployed during the year prior to the index suicide attempt (32.2% vs 19.8% respectively). A lower proportion of refugees received sickness absence benefits or disability pension (Table 1). The differences in the distribution of socio-demographic and labour market marginalisation factors among the Swedish-born and refugees were all statistically significant (Table 1, *p* < 0.05).

### Characteristics related to index suicide attempt

A lower proportion of refugees had a history of hospitalisation or visit to specialised outpatient healthcare due to suicide attempt, compared with the Swedish-born (Supplementary Table 1). Compared to Swedish-born, refugees were more often diagnosed with depressive, anxiety or post-traumatic stress disorders (13.3% vs 9.1%). All the differences in the distributions of characteristics regarding index suicide attempt among the Swedish-born and refugees were statistically significant (Supplementary Table 1, *p* < 0.0001).

### Prevalence of inpatient psychiatric healthcare

**Fig. 1:**
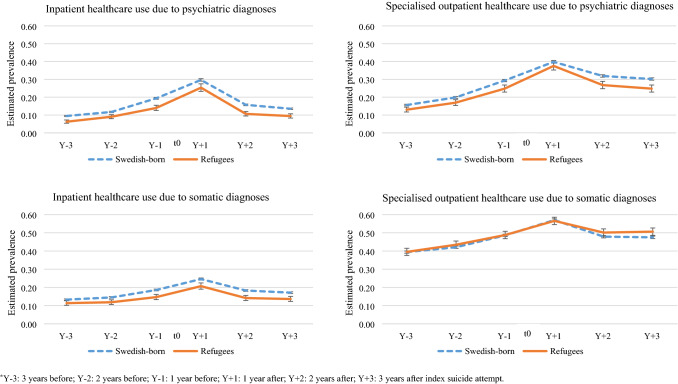
Estimated annual prevalence of specialised psychiatric and somatic healthcare use, adjusted for sex, age, educational level and year of index suicide attempt, at different time points* 3 years before and after seeking inpatient or specialised outpatient healthcare due to a suicide attempt (index suicide attempt) in between 2004 and 2013 (error bars indicate 95% confidence intervals)

Patterns of inpatient psychiatric healthcare were similar among the refugees and the Swedish-born. In both groups, adjusted prevalence rates of such healthcare increased from Y − 3 to Y + 1 and then decreased sharply in Y + 2 (Fig. 1). The crude prevalence of inpatient psychiatric healthcare showed that during the entire observation period, refugees had lower level of inpatient psychiatric healthcare than the Swedish-born and these differences were statistically significant (Table 2, *p* < 0.05). Similar patterns were also observed in the adjusted analyses (Fig. 1).

**Table 2:** Psychiatric healthcare use at different time points in terms of annual crude prevalence, mean number and duration (days) of hospitalisations and mean number of visits to specialised outpatient healthcare among 81,916 Swedish-born and 3855 refugees^a^, aged 20–64 years and residing in Sweden in the baseline year^b^ who sought inpatient or specialised outpatient healthcare due to a suicide attempt (index suicide attempt) in between 2004 and 2013

Time points^c^	Inpatient psychiatric healthcare use						Specialised outpatient psychiatric healthcare use			
	Number of individuals, *n* (%)		Mean number of hospitalisations among those with such care, *n* (sd)		Mean duration (days) of hospitalisation among those with such care, *n* (sd)		Number of individuals, *n* (%)		Mean number of visits among those with such care, *n* (sd)	
	Swedish-born	Refugees	Swedish-born	Refugees	Swedish-born	Refugees	Swedish-born	Refugees	Swedish-born	Refugees
Y − 3	**6849 (8.4)**	**231 (6.0)**	2.3 (2.4)	2.3 (2.3)	25.8 (43.6)	31.1 (42.7)	**11,661 (14.2)**	**483 (12.5)**	3.0 (3.6)	3.0 (3.2)
Y − 2	**8475 (10.3)**	**333 (8.6)**	2.4 (2.4)	2.2 (2.1)	27.4 (46.2)	28.4 (51.7)	**14,807 (18.1)**	**628 (16.3)**	3.3 (3.9)	3.5 (3.4)
Y − 1	**14,049 (17.2)**	**514 (13.3)**	2.4 (2.4)	2.3 (2.7)	27.3 (43.5)	26.8 (46.0)	**21,831 (26.7)**	**918 (23.8)**	3.7 (4.2)	3.6 (3.6)
Y + 1	**21,649 (26.4)**	**932 (24.2)**	**2.5 (2.7)**	**2.1 (2.4)**	32.0 (49.4)	29.9 (49.9)	29,672 (36.2)	1,389 (36.0)	**4.2 (4.6)**	**3.7 (3.7)**
Y + 2^d^	**11,094 (13.8)**	**387 (10.2)**	**2.8 (3.0)**	**2.4 (2.3)**	30.9 (51.3)	35.2 (61.5)	**23,276 (29.0)**	**981 (25.8)**	3.9 (4.5)	3.7 (3.6)
Y + 3^e^	**9391 (11.9)**	**339 (9.0)**	**1.7 (3.1)**	**1.3 (2.4)**	21.5 (51.6)	24.5 (65.2)	**21,693 (27.4)**	**900 (23.9)**	**3.0 (4.6)**	**2.7 (3.6)**

Bold text indicates a statistically significant difference between the Swedish-born individuals and the refugees with a *p *value < 0.05

^a^Individuals who settled in Sweden as ‘refugee’ or ‘in need of protection’ or, ‘humanitarian grounds’

^b^The year prior to the index suicide attempt

^c^Y − 3: 3 years before; Y − 2: 2 years before; Y − 1: 1 year before; Y + 1: 1 year after; Y + 2: 2 years after; Y + 3: 3 years after index suicide attempt

^d^Due to death or emigration during Y + 1, *n* = 1752 individuals (of which 38 refugees) were considered as drop-out during Y + 2

^e^Due to death or emigration during Y + 1 or Y + 2, *n* = 2917 individuals (of which 80 refugees) were considered as drop-out during Y + 3

### Prevalence of specialised outpatient psychiatric healthcare

Following the index suicide attempt, 36% Swedish-born and the same proportion of refugees were using outpatient psychiatric healthcare during Y + 1. The crude prevalence estimates declined slightly in the next two follow-up years in the Swedish-born but declined to a greater extent in refugees (Table 2). Adjusted prevalence of specialised outpatient psychiatric healthcare use had similar patterns in refugees and the Swedish-born—a sharp increase from Y − 3 to Y + 1 was followed by a steady decrease in both groups (Fig. 1). During all of the six observation years, refugees had lower adjusted prevalence of specialised outpatient healthcare due to psychiatric diagnoses, compared with the Swedish-born (Fig. 1).

### Psychiatric healthcare visits and duration of hospitalisation

Refugees’ mean number of specialised healthcare visits due to a psychiatric diagnosis, before the index suicide attempt did not differ from that of the Swedish-born (Table 2). On the other hand, specialised psychiatric healthcare visits after the index suicide attempt, were generally fewer in refugees than the Swedish-born, both in inpatient and specialised outpatient healthcare (Table 2). Both before and after the index suicide attempt, there were no significant differences in psychiatric healthcare use, in terms of the mean duration (days) of hospitalisation, between the Swedish-born and the refugees. Nonetheless, the point estimates showed that refugees generally had higher mean duration of hospitalisation than the Swedish-born during the follow-up (Table 2).

### Prevalence of inpatient somatic healthcare

Crude prevalence of inpatient somatic healthcare use in refugee suicide attempters was generally lower during all the six observation years than the Swedish-born suicide attempters and these differences were statistically significant (Table 3, *p* < 0.05). The highest crude prevalence for both groups was during Y + 1 when 22.2% Swedish-born and 18.6% refugees received inpatient somatic healthcare. Although the crude prevalence of healthcare use declined following Y + 1, the levels at Y + 3 were still higher than the levels at Y − 3 (Table 3).

**Table 3:** Somatic healthcare use at different time points in terms of annual crude prevalence, mean number and duration (days) of hospitalisations and mean number of visits to specialised outpatient healthcare among 81,916 Swedish-born and 3855 refugees^a^, aged 20–64 years and residing in Sweden in the baseline year^b^ who sought inpatient or specialised outpatient healthcare due to a suicide attempt (index suicide attempt) in between 2004 and 2013

Time points^c^	Inpatient somatic healthcare use						Specialised outpatient somatic healthcare use			
	Number of individuals, *n* (%)		Mean number of hospitalisations among those with such care, *n* (sd)		Mean duration (days) of hospitalisation among those with such care, *n* (sd)		Number of individuals, *n* (%)		Mean number of visits among those with such care, *n* (sd)	
	Swedish-born	Refugees	Swedish-born	Refugees	Swedish-born	Refugees	Swedish-born	Refugees	Swedish-born	Refugees
Y − 3	**9797 (12.0)**	**392 (10.2)**	**1.7 (1.7)**	**1.4 (1.0)**	**7.7 (15.0)**	**5.2 (7.6)**	29,895 (36.5)	1424 (36.9)	2.5 (2.9)	2.5 (4.1)
Y − 2	**10,673 (13.0)**	**409 (10.6)**	**1.8 (2.0)**	**1.5 (1.3)**	**8.4 (15.3)**	**6.6 (12.9)**	31,998 (39.1)	1569 (40.7)	2.6 (3.0)	2.5 (2.4)
Y − 1	**13,698 (16.7)**	**507 (13.2)**	**1.9 (2.1)**	**1.6 (1.4)**	**8.9 (16.8)**	**6.7 (11.7)**	37,146 (45.3)	1769 (45.9)	**2.8 (3.4)**	**2.7 (2.7)**
Y + 1	**18,156 (22.2)**	**718 (18.6)**	**1.9 (2.0)**	**1.6 (1.2)**	10.3 (21.2)	9.3 (24.7)	43,940 (53.6)	2065 (53.6)	**3.0 (3.6)**	**2.9 (2.9)**
Y + 2^d^	**13,123 (16.4)**	**482 (12.7)**	**2.0 (2.0)**	**1.5 (1.2)**	**8.4 (15.4)**	**6.2 (10.6)**	**35,868 (44.7)**	**1801 (47.3)**	**2.9 (3.4)**	**2.7 (2.6)**
Y + 3^e^	**12,068 (15.3)**	**459 (12.2)**	**1.0 (2.0)**	**0.6 (1.4)**	**4.8 (14.1)**	**2.3 (7.8)**	**35,023 (44.3)**	**1803 (47.8)**	**2.1 (3.7)**	**2.0 (2.8)**

Bold text indicates a statistically significant difference between the Swedish-born individuals and the refugees with a *p*-value < 0.05

^a^Individuals who settled in Sweden as 'refugee' or 'in need of protection' or, 'humanitarian grounds'

^b^The year prior to the index suicide attempt

^c^Y − 3: 3 years before; Y − 2: 2 years before; Y − 1: 1 year before; Y + 1: 1 year after; Y + 2: 2 years after; Y + 3: 3 years after index suicide attempt

^d^Due to death or emigration during Y + 1, *n* = 1752 individuals (of which 38 refugees) were considered as drop-out during Y + 2

^e^Due to death or emigration during Y + 1 or Y + 2, *n* = 2917 individuals (of which 80 refugees) were considered as drop-out during Y + 3

Similar patterns of inpatient somatic healthcare use were observed among the refugees and the Swedish-born, i.e. in both groups, adjusted prevalence of somatic healthcare use increased from Y − 3, reached peak at Y + 1 and then steadily decreased in Y + 2 and Y + 3 (Fig. 1). During all of the observation years, refugees had lower adjusted prevalence of inpatient somatic healthcare use than the Swedish-born (Fig. 1).

### Prevalence of specialised outpatient somatic healthcare

Crude prevalence of specialised outpatient somatic healthcare use among the Swedish-born and the refugees were similar during Y + 1 but slightly higher among the refugees during Y + 2 and Y + 3 (Table 3, *p* < 0.05). Adjusted prevalence of specialised outpatient healthcare use due to somatic diagnoses followed slightly different patterns in refugees than the Swedish-born. Although, for both groups a similar sharp increase was observed until Y + 1, the decrease in healthcare use from Y + 1 to Y + 2 was more prominent among the Swedish-born than the refugees (Fig. 1). It is also shown that refugees had higher adjusted prevalence of specialised outpatient somatic healthcare use during Y + 2 and Y + 3 than the Swedish-born (Fig. 1). Approximately 47% Swedish-born and 51% refugees received specialised outpatient somatic healthcare during Y + 3, and these multivariate-adjusted prevalence estimates differed significantly (Fig. 1).

### Somatic healthcare visits and duration of hospitalisation

Refugees, compared with the Swedish-born, generally had lower inpatient somatic healthcare use in terms of both mean number and mean duration (days) of hospitalisations, both before and after the index suicide attempt (Table 3). The mean number of healthcare visits to specialised outpatient did not differ between the groups during Y − 2 and Y − 3, but otherwise, was lower in refugees during the rest of the observation years, in comparison with the Swedish-born (Table 3).

### Determinants of all-cause specialised healthcare use in refugees

**Fig. 2:**
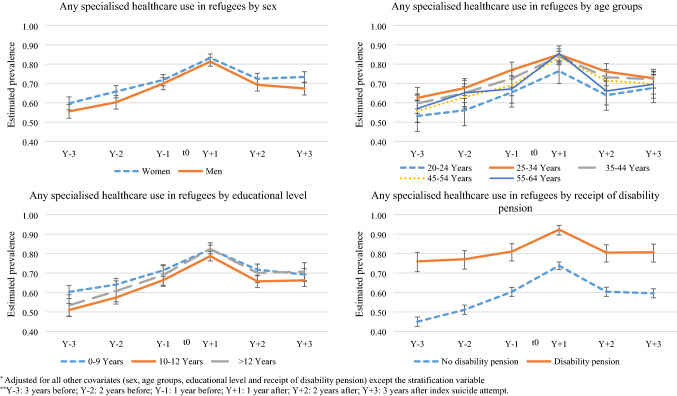
Estimated annual adjusted* prevalence of any specialised (inpatient or specialised outpatient) healthcare use in refugees, stratified by sex, age, educational level and receipt of disability pension, at different time points** 3 years before and after seeking inpatient or specialised outpatient healthcare due to a suicide attempt (index suicide attempt) in between 2004 and 2013 (error bars indicate 95% confidence intervals)

In the crude models, specialised healthcare use among refugees differed by sex, age and according to receipt of disability pension but not by educational level (data not shown). In the adjusted models, specialised healthcare use among refugees differed only according to receipt of disability pension (Fig. 2). A significantly higher proportion of refugees who were disability pension recipients at index suicide attempt used specialised healthcare during all of the observation years than refugees who did not receive disability pension (Fig. 2). Among the refugees who did not receive disability pension at baseline, adjusted prevalence of healthcare use rose sharply from Y − 3 to Y + 1 (from 45 to 74%) and then dropped sharply in Y + 2 (60%). In contrast, refugees with disability pension only used specialised healthcare to a greater extent during Y + 1. For rest of the observation years, their adjusted prevalence of specialised healthcare use remained somewhat constant (Fig. 2).

## Discussion

### Main findings

In this nationwide open cohort of 85,771 index suicide attempters seeking specialised healthcare during 2004–2013 in Sweden, we found a lower crude and adjusted prevalence of psychiatric and somatic healthcare use in refugees during an observation window of 3 years before and 3 years after the index suicide attempt, compared with Swedish-born individuals. Higher specialised healthcare use was observed among refugees receiving disability pension at the index suicide attempt, compared with disability pension non-recipients.

### Patterns of healthcare use in refugees

We found a lower specialised healthcare use in refugees, in general, compared with the Swedish-born. However, the patterns were somewhat similar during the 6 observation years in both groups, i.e. increase in healthcare use until Y + 1 and then decrease. Such patterns of healthcare use were expected as previous studies in general populations showed an increased use of healthcare services during the years before and after a suicide attempt [38, 39]. Our study showed that the same pattern holds also for a cohort of refugee suicide attempters.

### Specialised psychiatric and somatic healthcare use

Both crude and adjusted prevalence rates of specialised psychiatric healthcare use were lower in refugees, compared with the Swedish-born in this study. Due to the lack of similar previous studies, direct comparisons are not possible. However, these results can be considered to be comparable with studies that reported lower psychiatric aftercare following a suicide attempt [11]. Non-European immigrants to Europe received lower referral to specialised healthcare by healthcare professionals after a suicide attempt [14] and south Asian migrants were less likely to be offered specialist psychiatric health services following self-harm in the UK, than the majority population [15].

A lower inpatient somatic healthcare use in refugees, in comparison with the Swedish-born was also observed. On the other hand, the adjusted prevalence of specialised outpatient somatic healthcare use did not differ between the refugees and the Swedish-born during Y − 3 to Y + 1. Also, specialised outpatient somatic healthcare use, in terms of the mean number of visits, did not differ between the two groups. It is also difficult to explain the slightly higher adjusted prevalence of specialised outpatient somatic healthcare use in refugees during Y + 3. Somatisation was explained as idioms of distress in refugee populations before [23]. Moreover, cultural unfamiliarity with symptoms of mental ill-health, lack of confidence on host-country’s mental healthcare system or stigma towards mental illness [9] may influence the refugees to express their mental health issues as somatic problems. All these factors might have contributed to over or underestimation of specialised psychiatric healthcare use in refugees and may have led to these contradictory findings. As no previous study has investigated somatic healthcare use in refugees or immigrants following a suicide attempt, direct comparisons could not be drawn. Previously, only male refugees were found to be more likely to use primary healthcare in Canada than in long-term residents [26]. Future studies including primary healthcare use in refugees may help explain this phenomenon.

### Healthcare use before and after a suicide attempt

Although the crude and adjusted prevalence of healthcare use was generally lower in refugees, compared with the Swedish-born, some deviations from this general trend are noteworthy. First of all, inpatient psychiatric healthcare, in terms of mean number of hospitalisations, did not differ between the groups. Moreover, duration of hospitalisation did not differ much between the refugees and Swedish-born healthcare users. Among those who had specialised healthcare, it seems like the difference in psychiatric healthcare use between these groups was not as prominent as the whole cohort. As the national health insurance system in Sweden ensures universal access to healthcare for everyone, limited access should not be a problem only for refugees. Still, several barriers to healthcare access, both on the receiver’s and the provider’s side may explain this phenomenon of lower healthcare use both before and after a suicide attempt in refugees.

Owing to language barriers, lack of information and inadequate knowledge of how the healthcare system works in Sweden, refugees may become less prone to seek healthcare than the Swedish-born. Furthermore, stigma towards mental ill-health and perceived negative experience with healthcare services [8] may make the refugees decline adequate aftercare following a suicide attempt which might be reflected in our results.

Barriers to healthcare access can also arise from the service provider’s side. Healthcare professionals may misinterpret the health problems expressed by the refugees because of language issues and cultural differences [8]. Also, clinicians may consider the suicidal behaviour in refugees as ‘less serious’ than the Swedish-born because they might view the suicide attempt in refugees as a ‘general’ response to prior traumatic life events and may overlook other reasons behind the suicidal behaviour. This may lead to inadequate referral following a suicide attempt and consequently, lower healthcare use in refugees. To overcome some of the barriers to healthcare access, more cultural competence among healthcare professionals may be necessary, especially when managing suicide attempters. Moreover, improving the health literacy among refugees and ensuring adequate aftercare may also diminish the gap in healthcare use between the Swedish-born and the refugees, both before and after a suicide attempt. Access to healthcare can also be negatively affected in refugees due to worry about healthcare expenses or if attending a healthcare visit is not possible due to a precarious job situation or due to childcare at home [9]. For such instances, social support and the welfare system may play a vital role to improve healthcare access.

Apart from the barriers to healthcare access, the “healthy migrant” hypothesis [18] may also explain the lower healthcare use in refugees in this study. That is, refugees in Sweden could be a selected group who, because of their better health, were able to undertake the physically and mentally demanding process of migration to Sweden [18]. Considering this hypothesis, one of the explanations underlying the finding of lower healthcare use in refugees could be that refugees sought healthcare services less because of their comparatively better health status. A study on the general population in Sweden [7] showed similar differences regarding morbidity factors at baseline between the Swedish-born and refugees. This may hint that if a positive selection exists in refugees belonging to the general population in Sweden, it probably also exists among the refugees who attempted suicide. Further investigation is required to understand these mechanisms in refugee suicide attempters.

### Determinants of healthcare use

We found higher specialised healthcare use in refugees receiving disability pension at baseline compared with the non-recipients. Receipt of disability pension in Sweden requires a temporal or permanent reduction of work capacity due to disease or injury [30]. Individuals below 30 years of age can also be granted disability pension if they have not completed compulsory education. Because of the disease or injury that led to the disability pension, these refugees were possibly in greater and more frequent need of healthcare services than any other group. Therefore, the pattern of specialised healthcare use in this group did not vary much during the observation period except for the year immediately after the index suicide attempt.

### Methodological considerations

The possibility to prospectively follow all refugees as well as the Swedish-born population, who were treated for suicide attempt in specialised healthcare, by linking several population-based nationwide high-quality registers [30, 31, 40, 41] is the main strength of this study. This study design allowed us to avoid recall bias regarding the measured variables and selection bias from non-response. Another strength is that we could consider several key variables in our analyses.

The study, however, has also limitations. First, we could only consider specialised healthcare use, which limits generalisability to healthcare use due to less severe forms of morbidity. Because national level primary healthcare data was not available, we could not compare such healthcare use patterns between the Swedish-born and refugees. Second, due to the nature of the data collection, i.e. register data, we could only include individuals who were treated for suicide attempt in specialised healthcare. According to surveys, only about half of the suicide attempts in Sweden receive inpatient healthcare [42]. Moreover, because the data related to the index suicide attempt originate from specialised healthcare, there could be more underreporting of suicide attempts among refugees due, for example, to stigma associated with suicidal behaviour. However, we think that we could diminish bias due to differential underreporting by including events of undetermined intent (ICD-10 codes: Y10–Y34) as suicide attempts in our study.

Additionally, a sensitivity analysis revealed that specialised healthcare generally did not differ between the Swedish-born and refugees if we restrict our cohort to only those without any baseline labour market marginalisation. This may indicate that the lower use of specialised healthcare in refugees, compared with the Swedish-born, is particularly found among refugees who are marginalised in the labour market. However, limited statistical power in this sensitivity analysis restricts any firm conclusion based on these results. Finally, in addition to the conventional definition of refugees, we have also included individuals in the refugee sample who were granted residence permit on ‘in need of protection’ or ‘humanitarian grounds’. This way, a negative health selection among our refugee population might be possible, because individuals granted residence permit on ‘humanitarian grounds’ in Sweden were found to be less healthy than any other groups [43]. However, similar healthcare use patterns were observed in our sensitivity analyses, including and excluding these individuals (data not shown).

## Conclusion

Our study revealed that during a period of 3 years before and 3 years after the index suicide attempt, refugees generally used specialised healthcare less than the Swedish-born population. Among refugee suicide attempters, higher specialised healthcare use was observed among disability pension recipients compared with non-recipients. Future research, preferably qualitative in nature, should focus on finding specific mechanisms behind the underutilisation of healthcare in refugee suicide attempters, so that specific measures can be taken to improve healthcare use in this vulnerable group.

## Electronic supplementary material

Below is the link to the electronic supplementary material.

Supplementary file 1 (DOCX 15 kb)

Supplementary file 2 (DOCX 209 kb)

## Abbreviations

CI: Confidence interval
GEE: Generalized estimating equations

## References

[1] Hawton K, Saunders KE, O'Connor RC (2012). Self-harm and suicide in adolescents. Lancet.

[2] Tinghög P, Malm A, Arwidson C, Sigvardsdotter E, Lundin A, Saboonchi F (2017). Prevalence of mental ill health, traumas and postmigration stress among refugees from Syria resettled in Sweden after 2011: a population-based survey. BMJ Open.

[3] Lindert J, Ehrenstein OS, Priebe S, Mielck A, Brähler E (2009). Depression and anxiety in labor migrants and refugees—a systematic review and meta-analysis. Soc Sci Med.

[4] Hawton K, Casanas ICC, Haw C, Saunders K (2013). Risk factors for suicide in individuals with depression: a systematic review. J Affect Disord.

[5] Bentley KH, Franklin JC, Ribeiro JD, Kleiman EM, Fox KR, Nock MK (2016). Anxiety and its disorders as risk factors for suicidal thoughts and behaviors: a meta-analytic review. Clin Psychol Rev.

[6] Bursztein Lipsicas C, Makinen IH, Apter A, De Leo D, Kerkhof A, Lonnqvist J, Michel K, Salander Renberg E, Sayil I, Schmidtke A, van Heeringen C, Varnik A, Wasserman D (2012). Attempted suicide among immigrants in European countries: an international perspective. Soc Psychiatry Psychiatr Epidemiol.

[7] Amin R, Helgesson M, Runeson B, Tinghög P, Mehlum L, Qin P, Holmes EA, Mittendorfer-Rutz E (2019). Suicide attempt and suicide in refugees in Sweden—a nationwide population-based cohort study. Psychol Med.

[8] Lindert J, Schouler-Ocak M, Heinz A, Priebe S (2008). Mental health, health care utilisation of migrants in Europe. Eur Psychiatry.

[9] Van Der Boor CF, White R (2020). Barriers to accessing and negotiating mental health services in asylum seeking and refugee populations: the application of the candidacy framework. J Immigr Minor Health.

[10] Tinghög P (2009) Migration, stress and mental ill health: post-migration factors and experiences in the Swedish context. Linköping University

[11] Sundvall M, Tidemalm DH, Titelman DE, Runeson B, Bäärnhielm S (2015). Assessment and treatment of asylum seekers after a suicide attempt: a comparative study of people registered at mental health services in a Swedish location. BMC Psychiatry.

[12] Hjern A (2001). High use of sedatives and hypnotics in ethnic minorities in Sweden. Ethn Health.

[13] Lawrence RE, Oquendo MA, Stanley B (2016). Religion and suicide risk: a systematic review. Arch Suicide Res.

[14] Bursztein Lipsicas C, Makinen IH, Wasserman D, Apter A, Bobes J, Kerkhof A, Michel K, Renberg ES, van Heeringen K, Varnik A, Schmidtke A (2014). Immigration and recommended care after a suicide attempt in Europe: equity or bias?. Eur J Public Health.

[15] Cooper J, Husain N, Webb R, Waheed W, Kapur N, Guthrie E, Appleby L (2006). Self-harm in the UK: differences between South Asians and Whites in rates, characteristics, provision of service and repetition. Soc Psychiatry Psychiatr Epidemiol.

[16] Bogic M, Njoku A, Priebe S (2015). Long-term mental health of war-refugees: a systematic literature review. BMC Int Health Hum Rights.

[17] McCrone P, Bhui K, Craig T, Mohamud S, Warfa N, Stansfeld SA, Thornicroft G, Curtis S (2005). Mental health needs, service use and costs among Somali refugees in the UK. Acta Psychiatr Scand.

[18] Abebe DS, Lien L, Elstad JI (2017). Immigrants' utilization of specialist mental healthcare according to age, country of origin, and migration history: a nation-wide register study in Norway. Soc Psychiatry Psychiatr Epidemiol.

[19] Elstad JI (2016). Register study of migrants' hospitalization in Norway: world region origin, reason for migration, and length of stay. BMC Health Serv Res.

[20] Sarria-Santamera A, Hijas-Gomez AI, Carmona R, Gimeno-Feliu LA (2016). A systematic review of the use of health services by immigrants and native populations. Public Health Rev.

[21] Graetz V, Rechel B, Groot W, Norredam M, Pavlova M (2017). Utilization of health care services by migrants in Europe—a systematic literature review. Br Med Bull.

[22] National Board of Health and Welfare (2020) Suicid. Suicide (In Swedish). https://patientsakerhet.socialstyrelsen.se/risker/vardskadeomraden/suicid. Accessed 19 Mar 2020

[23] Rohlof HG, Knipscheer JW, Kleber RJ (2014). Somatization in refugees: a review. Soc Psychiatry Psychiatr Epidemiol.

[24] Money TT, Batterham PJ (2019). Sociocultural factors associated with attitudes toward suicide in Australia. Death Stud.

[25] Nielsen SS, Jensen NK, Kreiner S, Norredam M, Krasnik A (2015). Utilisation of psychiatrists and psychologists in private practice among non-Western labour immigrants, immigrants from refugee-generating countries and ethnic Danes: the role of mental health status. Soc Psychiatry Psychiatr Epidemiol.

[26] Durbin A, Lin E, Moineddin R, Steele LS, Glazier RH (2014). Use of mental health care for nonpsychotic conditions by immigrants in different admission classes and by refugees in Ontario, Canada. Open Med.

[27] Helgesson M, Wang M, Niederkrotenthaler T, Saboonchi F, Mittendorfer-Rutz E (2019). Labour market marginalisation among refugees from different countries of birth: a prospective cohort study on refugees to Sweden. J Epidemiol Community Health.

[28] UNHCR (2019) Global trends—forced displacement in 2018. Geneva

[29] Statistics Sweden (SCB) Review of previously published statistics regarding reason for residence (in Swedish). https://www.scb.se/contentassets/9171f415739b4211addb78298247d3bc/oversyn-av-tidigare-publicerad-statistik-grund-for-bosattning.pdf. Accessed 20 Feb 2019

[30] Ludvigsson JF, Svedberg P, Olén O, Bruze G, Neovius M (2019). The longitudinal integrated database for health insurance and labour market studies (LISA) and its use in medical research. Eur J Epidemiol.

[31] Brooke HL, Talbäck M, Hörnblad J, Johansson LA, Ludvigsson JF, Druid H, Feychting M, Ljung R (2017). The Swedish cause of death register. Eur J Epidemiol.

[32] Linsley KR, Schapira K, Kelly TP (2001). Open verdict v. suicide—importance to research. Br J Psychiatry.

[33] Runeson B, Haglund A, Lichtenstein P, Tidemalm D (2015). Suicide risk after nonfatal self-harm: a national cohort study, 2000–2008. J Clin Psychiatry.

[34] Mittendorfer Rutz E, Wasserman D (2004). Trends in adolescent suicide mortality in the WHO European Region. Eur Child Adolesc Psychiatry.

[35] Nock MK, Borges G, Bromet EJ, Cha CB, Kessler RC, Lee S (2008). Suicide and suicidal behavior. Epidemiol Rev.

[36] Cheng A, Fremont C (2006) Duration calculation from a clinical programmer’s perspective. https://support.sas.com/resources/papers/proceedings/proceedings/sugi31/048-31.pdf. Accessed 10 Oct 2019

[37] Ballinger GA (2004). Using generalized estimating equations for longitudinal data analysis. Organ Res Methods.

[38] Suominen KH, Isometsa ET, Ostamo AI, Lonnqvist JK (2002). Health care contacts before and after attempted suicide. Soc Psychiatry Psychiatr Epidemiol.

[39] Michel K, Runeson B, Valach L, Wasserman D (1997). Contacts of suicide attempters with GPs prior to the event: a comparison between Stockholm and Bern. Acta Psychiatr Scand.

[40] Ludvigsson JF, Andersson E, Ekbom A, Feychting M, Kim JL, Reuterwall C, Heurgren M, Olausson PO (2011). External review and validation of the Swedish national inpatient register. BMC Public Health.

[41] Forsberg L, Rydh H, Björkenstam E, Jacobsson A, Nyqvist K, Heurgren M (2009) Kvalitet och innehåll i patientregistret. Utskrivningar från slutenvården 1964–2007 och besök i specialiserad öppenvård (exklusive primärvårdsbesök) 1997–2007. (Quality and content of the Patient Register. Discharges from inpatient care in 1964–2007 and visits to specialised outpatient care (excluding primary care visits) in 1997–2007.) (in Swedish). Socialstyrelsen

[42] National Centre for Suicide Research and Prevention of Mental lll-Health (NASP) (2019) Hur beräknas självmordsstatistik? How statistics on suicidal behaviour are calculated (In Swedish)? https://ki.se/nasp/hur-beraknas-sjalvmordsstatistik. Accessed 24 Oct 2019

[43] Hollander AC, Bruce D, Burström B, Ekblad S (2011). Gender-related mental health differences between refugees and non-refugee immigrants-a cross-sectional register-based study. BMC Public Health.

