# Supplementary material for: Healthcare use before and after suicide attempt in refugees and Swedish-born individuals
Source: Soc Psychiatry Psychiatr Epidemiol. 2020 Jun 16;56(2):325–38. doi: 10.1007/s00127-020-01902-z (PMC7870606; doi:10.1007/s00127-020-01902-z)
Supplement: Supplementary file 1 — (DOCX 15 kb) [file 127_2020_1902_MOESM1_ESM.docx]

| Supplementary table S1: Descriptive statistics of factors related to a suicide attempt (index suicide attempt) of 81,916 Swedish-born and 3,855 refugees^a^, aged 20-64 years and residing in Sweden in the baseline year^b^ who sought inpatient or specialised outpatient healthcare for the index suicide attempt in between 2004 and 2013 | | | |
| --- | --- | --- | --- |
| **Characteristics** | **All  n (%)** | **Swedish-born n (%)** | **Refugees n (%)** |
|  | 85,771 (100.0) | 81,916 (95.5) | 3,855 (4.5) |
| *History of suicide attempt^c^* |  |  |  |
| No | 73,641 (85.9) | 70,143 (85.6) | 3,498 (90.7) |
| Yes | 12,130 (14.1) | 11,773 (14.4) | 357 (9.3) |
| *Method of index suicide attempt* |  |  |  |
| Poisoning (X60-69, Y10-19)^d^ | 42,579 (49.6) | 40,433 (49.4) | 2,146 (55.7) |
| Hanging, strangulation and suffocation (X70, Y20)^d^ | 1,052 (1.2) | 992 (1.2) | 60 (1.6) |
| Firearm or explosives (X72-75, Y22-25)^d^ | 328 (0.4) | 293 (0.4) | 35 (0.9) |
| Cutting or piercing (X78, Y28)^d^ | 8,462 (9.9) | 8,065 (9.8) | 397 (10.3) |
| Jumping from a height (X80, Y30)^d^ | 1,079 (1.3) | 1,021 (1.2) | 58 (1.5) |
| Others (X71, X76-77, X79, X81-84, Y21, Y26-27, Y29, Y31-34)^d^ | 32,271 (37.6) | 31,112 (38.0) | 1,159 (30.1) |
| *Mental disorder*^e^ *at index suicide attempt* |  |  |  |
| No diagnosed mental disorder | 66,315 (77.3) | 63,268 (77.2) | 3,047 (79.0) |
| Depressive disorders (F32-F34)^d^ | 4,708 (5.5) | 4,423 (5.4) | 285 (7.4) |
| Bipolar disorders (F30-F31)^d^ | 720 (0.8) | 709 (0.9) | 11 (0.3) |
| Anxiety disorders (F38-F48 except F43.1)^d^ | 3,153 (3.7) | 2,961 (3.6) | 192 (5.0) |
| Post-traumatic stress disorder (F43.1)^d^ | 97 (0.1) | 62 (0.1) | 35 (0.9) |
| Other mental disorders (F01-F29, F50-F99)^d^ | 10,778 (12.6) | 10,493 (12.8) | 285 (7.4) |
| Differences between the Swedish-born individuals and the refugees regarding all factors related to the index suicide attempt were statistically significant based on Chi-square tests (p < 0.05).  ^a^Individuals who settled in Sweden as 'refugee' or 'in need of protection' or, 'humanitarian grounds'.  ^b^The year prior to the index suicide attempt.  ^c^Measured as any inpatient or specialised outpatient healthcare due to suicide attempt during 1987-2003 and 2001-2003 respectively  ^d^International Classification of Diseases version 10 code(s)  ^e^As main or side diagnosis in specialised healthcare. | | | |
