# Supplementary material for: Healthcare use before and after suicide attempt in refugees and Swedish-born individuals
Source: Soc Psychiatry Psychiatr Epidemiol. 2020 Jun 16;56(2):325–38. doi: 10.1007/s00127-020-01902-z (PMC7870606; doi:10.1007/s00127-020-01902-z)
Supplement: Supplementary file 2 — (DOCX 209 kb) [file 127_2020_1902_MOESM2_ESM.docx]

| **Supplementary Fig. 1** Estimated prevalence of specialised psychiatric and somatic healthcare use in refugees and the Swedish-born population who had no unemployment or sickness absence or disability pension in the baseline year, adjusted for sex, age, educational level and year of index suicide attempt, at different time points^*^ three years before and after seeking inpatient or specialised outpatient healthcare due to a suicide attempt (index suicide attempt) in between 2004 and 2013 (error bars indicate 95% Confidence Intervals) | |
| --- | --- |
| t0 | t0 |
| t0 | t0 |
| ^*^Y-3: 3 years before; Y-2: 2 years before; Y-1: 1 year before; Y+1: 1 year after; Y+2: 2 years after; Y+3: 3 years after index suicide attempt. | |
